# Coronary obstruction analysis in transcatheter aortic valve implantation through patient-specific computational modelling

**DOI:** 10.3389/fcvm.2024.1432235

**Published:** 2024-10-17

**Authors:** Jiaqi Fan, Jun Chen, Lihan Wang, Po Hu, Jubo Jiang, Xinping Lin, Giorgia Rocatello, Matthieu De Beule, Yi Tie, Yifei Wang, Sihang Cheng, Xianbao Liu, Jian’an Wang

**Affiliations:** ^1^ Department of Cardiology, Second Affiliated Hospital Zhejiang University School of Medicine, Hangzhou, China; ^2^ Department of Epidemiology, Harvard T.H. Chan School of Public Health, Boston, MA, United States; ^3^ FEops NV, Gent, Belgium; ^4^ Venus Medtech, Hangzhou, China; ^5^ Zhejiang University School of Medicine, Hangzhou, China

**Keywords:** coronary obstruction, patient-specific computational simulation, transcatheter aortic valve replacement, prediction model, finite element mechanical analysis

## Abstract

**Background:**

Coronary obstruction (CO) is a rare but devasting complication during transcatheter aortic valve replacement (TAVR).

**Objectives:**

We aim to demonstrate that the predicted distance between the coronary ostia and the closest structure derived with patient-specific computer simulation is associated with CO risk during TAVR.

**Methods:**

We retrospectively analysed 14 aortic stenosis patients who underwent TAVR through finite element simulation. The frame deformation predicted with patient-specific computer simulation was qualitatively and quantitatively compared to the post-operative device deformation. The minimum distance between each coronary ostium and the closest structure was calculated and compared in patients who developed CO, at high risk of CO, and at no risk of CO.

**Results:**

Four patients experienced CO during TAVR, 5 patients were at high risk of CO, and the remaining 5 patients had no risk of CO. A high coefficient of determination was obtained for all measurements extracted from the simulated device and the post-operative device (≥0.95). Simulations predicted shorter distance between the coronary ostium and the closest structure in patients who experienced CO, compared to patients at high risk of CO or who did not experience this complication (right coronary: 5.9 vs. 6.8 vs. 8.8 mm, left coronary: 3.0 vs. 3.3 vs. 6.5 mm respectively).

**Conclusions:**

The distance between the coronary ostium and the closest structure was lower in patients who experienced CO during TAVR through patient-specific computational simulation. This technology enables coronary obstruction analysis before TAVR in the future.

## Introduction

Transcatheter aortic valve replacement (TAVR) has emerged as the recommended therapy for severe aortic stenosis patients aged over 65 years in the recent guideline (1). When performing TAVR on these patients, the reduction of periprocedural complications is of utmost importance. Among the complications following TAVR, coronary obstruction (CO) is rare (occurring in 0.7% of patients) but remains associated with a devasting outcome (2).

Most commonly CO occurs when the bulky native leaflet tissue is displaced over the coronary ostium during TAVR, with consequent obstruction of the coronary flow. CO can also occur when the native leaflet tissue is pushed towards the sinotubular junction, sealing completely the coronary sinuses (3). The risk factors for CO are very complex as they relate to the patient's anatomy, selected bioprosthesis (and its interaction with the surrounding anatomical structures), and procedural characteristics (2, 3). Also, previous studies reported weak predictors for CO with TAVR (2, 4, 5). Therefore, there is still a pressing unmet medical need for more sophisticated tool to identify patients who are in truly risk of CO with TAVR.

Traditional predictive methods often lack the precision needed to account for the intricate interactions between the aortic root's anatomical structures and the implanted valve, leading to variability in patient outcomes. Patient-specific computer simulations present a novel solution by combining baseline image-based anatomy with detailed biomechanical modeling of the aortic root and valve. These simulations, exemplified by tools like the FEops HEARTguide, have demonstrated significant accuracy in forecasting prosthesis deformation, calcium displacement, skirt sealing, and the risks of conduction abnormalities and paravalvular regurgitation (6–9). In this study we first verify the accuracy of the patient-specific simulation in terms of prediction of frame deformation with qualitive and quantitative comparison in a small cohort of patients. Then, we aim to demonstrate that the predicted distance between the coronary ostia and the closest structure (i.e., calcium nodule, native leaflet, implanted frame) derived with patient-specific computer simulation is associated with CO risk after TAVR.

## Methods

A retrospective single-center analysis was conducted on 14 patients with severe aortic stenosis who underwent transcatheter aortic valve replacement with a Venus A-Valve (Venus Medtech) with 4 patients underwent CO, 5 patients were at high risk of CO, and 5 patients were at low risk of CO. For all participants, CT imaging was performed both prior to and after the procedure (before discharge or at 30-day follow-up). Dual source computed tomography (DSCT) examinations were performed in the next generation CT (SOMATOM Definition Flash, Siemens Medical Solutions, Germany). All procedures were carried out as stated in earlier studies (9). The risk of CO was evaluated by JQ Fan based on the pre-procedural CT imaging. The occurrence of CO was confirmed by XB Liu based on the intraoperative coronary angiography.

The medical ethics committee of the Second Affiliated Hospital of Zhejiang University gave its approval, and the study was carried out in compliance with the Declaration of Helsinki's rules. For TAVR and the use of anonymized clinical, procedural, and follow-up data for research, each patient signed a written informed permission form.

### Patient-specific simulation

Preoperative DSCT were used to reconstruct the three-dimensional patient-specific aortic root anatomy for all patients, using the image segmentation software Mimics (Mimics v21.0, Materialise, Leuven, Belgium). The calcified native valve tissue was also included in the model.

**Figure 1:**
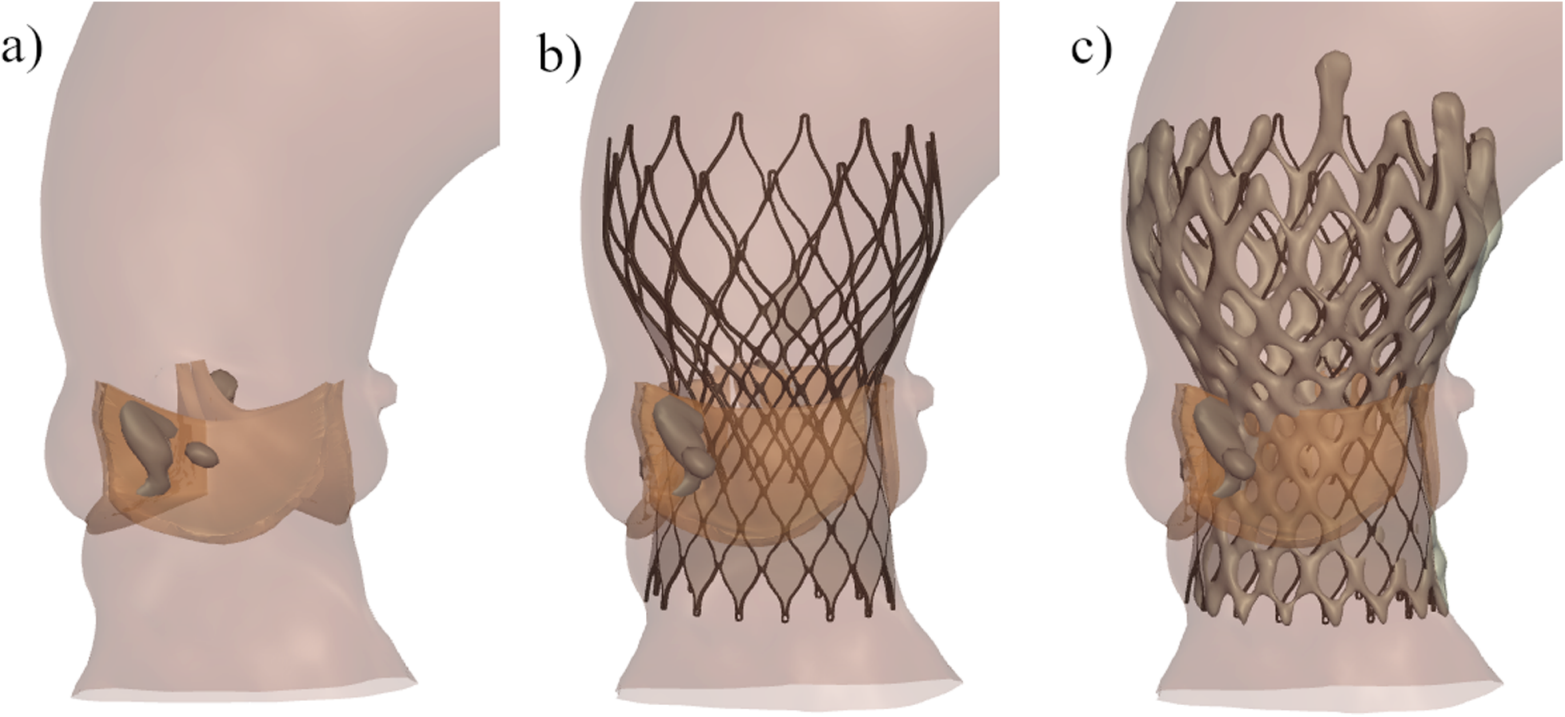
Representation of the main steps of the patient-specific computer simulation: **(a)** three-dimensional model of the aortic root anatomy including the native valve tissue (orange) and the calcium nodules (grey); **(b)** deployment of the Venus-A valve in the patients-specific aortic root; **(c)** overlay of the predicted frame (dark grey) and the reconstructed frame from postop CT (light grey).

Finite-element simulation performed with Abaqus (v6.12, Dassault Systemes, Simulia Corp, Johnson, RI) was used to reproduce the deployment of the Venus-A valve within the reconstructed aortic root (9). Details on the Venus-A valve model have been previously described (9). For each simulation, the selected valve size and device position were aligned with the clinical procedure. The simulation of the device deployment was iteratively adjusted until the simulated device position matched the actual device position, as derived from the postoperative DSCT (i.e., the post-operative geometry was reconstructed using Mimics and overlayed to the simulated geometry). All steps of the analysis are described in Figure 1.

### Frame deformation comparison

The post-operative device deformation was compared qualitatively and quantitatively to the predicted frame deformation for each patient. Qualitative comparison was performed by overlaying the predicted and post-operative devices. Quantitative comparison of frame dimensions was performed at four relevant device levels (i.e., commissures, central coaptation, nadir and ventricular end) in terms of minimum and maximum diameter, perimeter, and area (6, 9).

### Simulation-derived coronary occlusion analysis and statistical analysis

**Figure 2:**
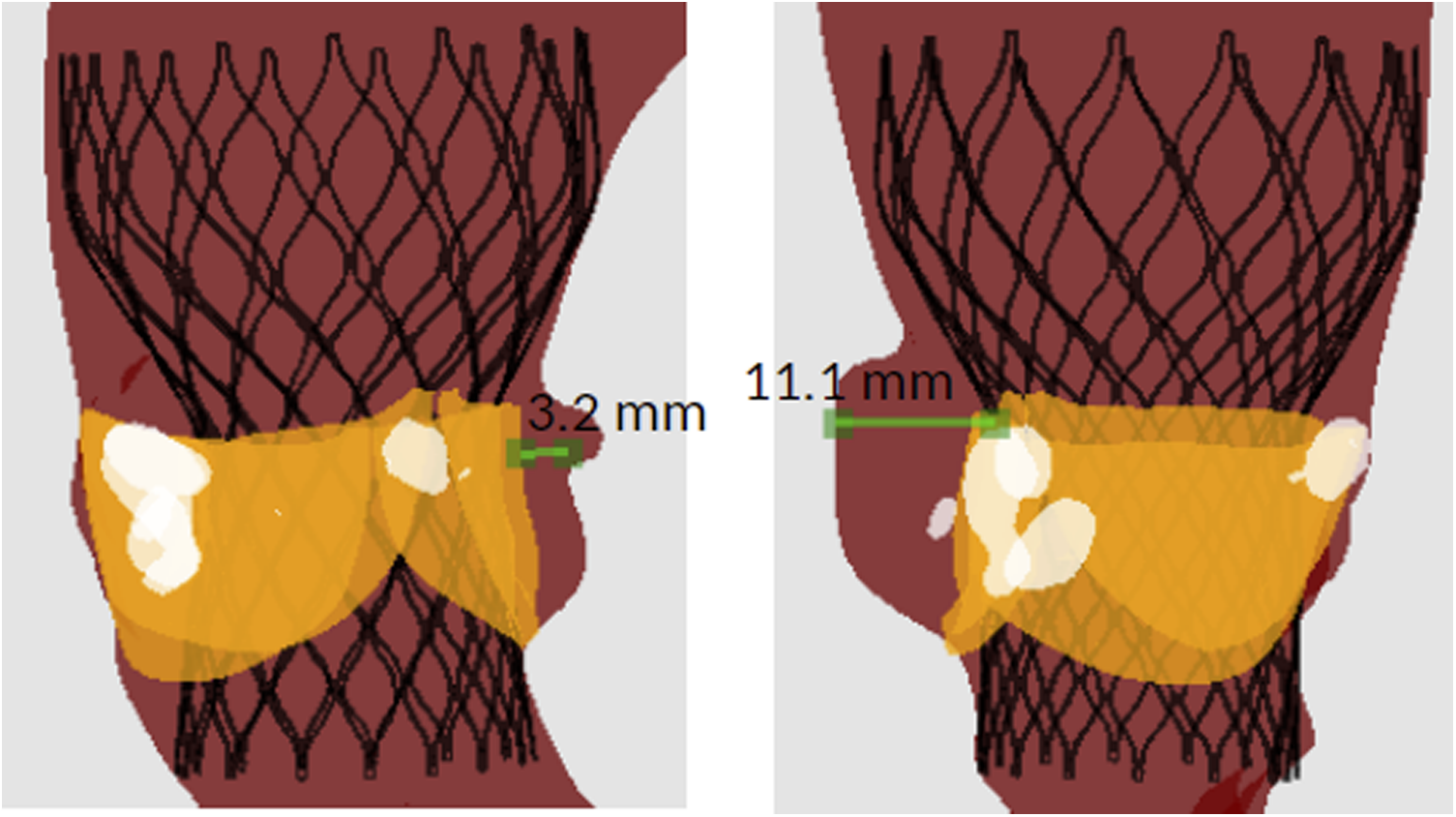
Example of the distance between the coronary ostium and the surrounding structures derived from simulation in one patient: (left) distance between the left coronary ostium and the native leaflet, (right) distance between the right coronary ostium and the calcium nodule.

After each finite-element simulation, the minimum distance between each coronary ostium and the closest structure (i.e., native valve leaflet, calcium nodule or Venus-A frame) was calculated, as shown in Figure 2.

Continuous variables are expressed as mean ± SD. Correlation between predicted and observed continuous variables was analysed using the coefficient of determination (R2). Comparison of the distance between the coronary ostium and closest prosthesis structure with clinical observation of the degree of coronary obstruction was carried out using box plot graphs. All graphs were generated with the Python module Matplotlib. Statistical analysis was performed with SciPy Stats, a Python module for probability functions and statistical distributions.

## Results

A total of 14 patients were included in this study. Four patients experienced CO during TAVR, 5 patients were at high risk of CO, while the remaining 5 patients had no risk of CO. In all patients with CO or at high risk of CO, the left coronary was affected. The baseline and procedural characteristics are summarized in Table 1. The height of STJ, RCA and LM seems to be lower in patients with CO.

**Table 1:** Baseline and procedural characteristics.

	Patients with CO (*n* = 4)	Patients at high risk of CO (*n* = 5)	Patients without CO (*n* = 5)
Age, years	73.50 ± 8.35	80.40 ± 5.55	71.60 ± 8.38
Male	1 (25.0)	3 (60.0)	5 (100.0)
BMI, kg/m^2^	23.98 ± 5.84	22.60 ± 2.62	22.40 ± 3.48
Smoker	0 (0.0)	0 (0.0)	3 (60.0)
Dyslipidemia	0 (0.0)	2 (40.0)	1 (20.0)
Hypertension	2 (50.0)	4 (80.0)	3 (60.0)
Diabetes mellitus	1 (25.0)	4 (80.0)	2 (40.0)
Syncope	1 (25.0)	1 (20.0)	0 (0.0)
NYHA.III.IV	4 (100.0)	4 (80.0)	4 (80.0)
STS score	11.10 ± 10.86	5.55 ± 2.28	5.09 ± 3.64
Prior MI	0 (0.0)	0 (0.0)	0 (0.0)
Prior PCI	1 (25.0)	0 (0.0)	1 (20.0)
Prior CABG	0 (0.0)	0 (0.0)	0 (0.0)
Prior stroke	0 (0.0)	0 (0.0)	1 (20.0)
Prior pacemaker	1 (25.0)	0 (0.0)	0 (0.0)
COPD	2 (50.0)	2 (40.0)	1 (20.0)
Pre-TTE data			
EF,%	60.60 ± 4.25	63.46 ± 11.19	53.28 ± 17.23
Maximum velocity, m/s	4.74 ± 0.46	5.25 ± 0.51	4.35 ± 0.84
Mean gradient, mmHg	55.25 ± 14.97	67.60 ± 13.58	42.20 ± 19.40
AVA, cm^2^	0.50 ± 0.17	0.74 ± 0.23	0.87 ± 0.35
AR moderate/severe	1 (25.0)	3 (60.0)	4 (80.0)
Pre-CT data			
Bicuspid aortic valve	1 (25.0)	1 (20.0)	2 (40.0)
Area, mm^2^	410.20 ± 98.99	445.98 ± 65.96	453.46 ± 113.92
Perimeter, mm	62.23 ± 28.63	76.46 ± 5.20	76.50 ± 9.88
STJ diameter, mm	27.52 ± 3.13	28.44 ± 2.40	31.32 ± 3.46
STJ height, mm	16.77 ± 1.34	19.12 ± 1.68	23.98 ± 3.06
Ascending aorta diameter at 4 cm, mm	38.42 ± 3.61	36.72 ± 2.48	34.66 ± 2.28
Maximum ascending aorta diameter, mm	42.67 ± 6.13	39.38 ± 3.42	37.00 ± 2.76
RCA height, mm	13.93 ± 0.69	15.38 ± 2.88	19.86 ± 1.78
LM height, mm	10.07 ± 1.76	13.48 ± 1.57	15.60 ± 2.36
Aortic root angle, degree	53.50 ± 7.94	50.80 ± 10.06	55.60 ± 11.55
Calcification grade			
None	0 (0.0)	0 (0.0)	1 (20.0)
Mild	1 (25.0)	0 (0.0)	1 (20.0)
Moderate	3 (75.0)	2 (40.0)	0 (0.0)
Severe	0 (0.0)	3 (60.0)	3 (60.0)
Procedural characteristics			
THV size, mm	25.25 ± 2.87	25.40 ± 1.34	26.60 ± 1.34
Predilation	4 (100.0)	5 (100.0)	4 (80.0)
Postdilation	1 (25.0)	2 (40.0)	4 (80.0)
Duration of procedure, min	172.33 ± 103.37	65.60 ± 21.30	61.60 ± 19.31

Values are mean ± SD or *n* (%).

AR, aortic regurgitation; AVA, aortic valve area; BMI, body mass index; CABG, coronary artery bypass graft; CO, coronary obstruction; COPD, chronic obstructive pulmonary disease; CT, computed tomography; EF, ejection fraction; LM, left main artery; MI, myocardial infarction; NYHA, New York Heart Association; PCI, percutaneous coronary intervention; RCA, right coronary artery; STJ, sinotubular junction; STS, society of thoracic surgeons; THV, transcatheter heart valve; TTE, transthoracic echocardiography.

### Frame deformation analysis

**Figure 3:**
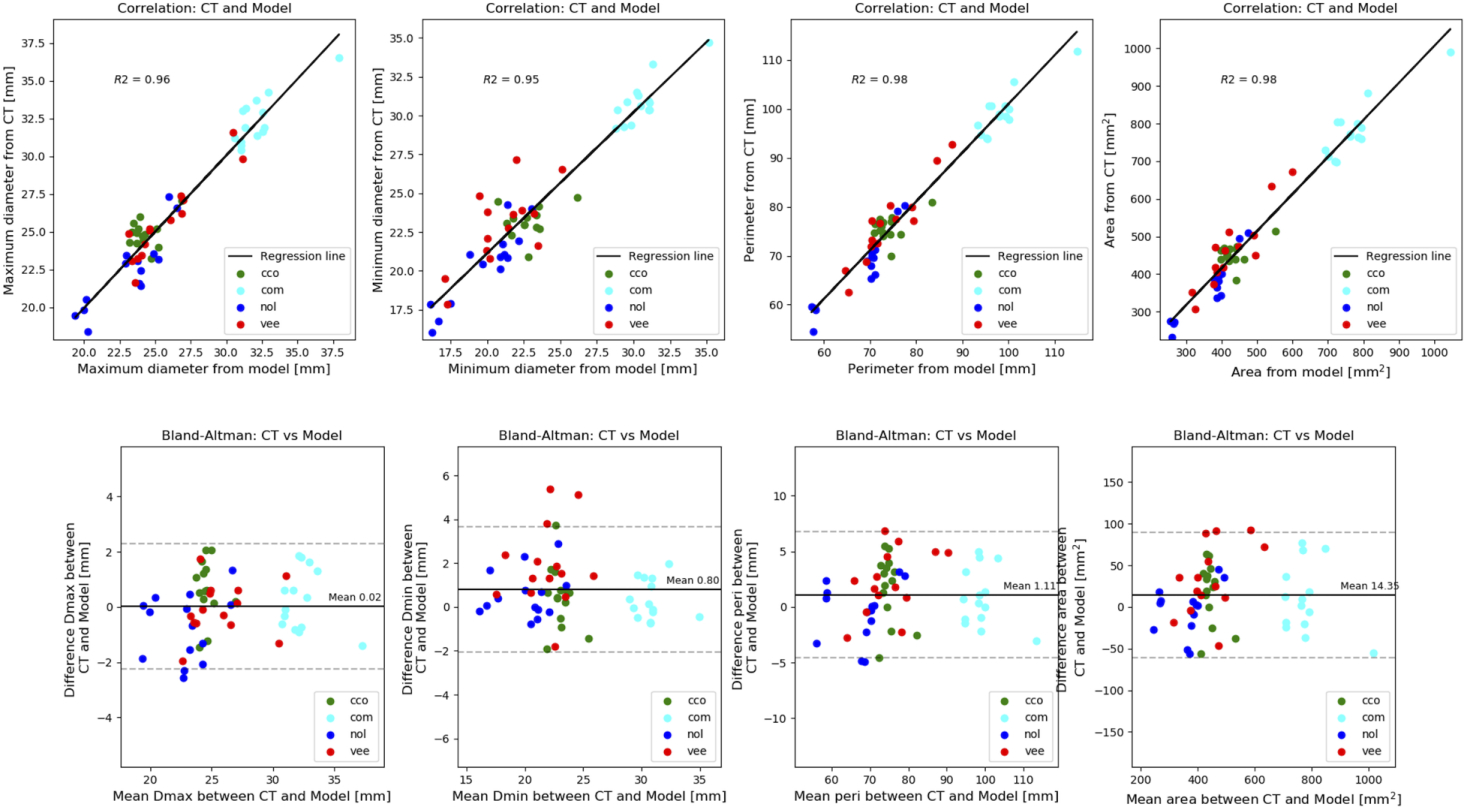
Comparison of frame deformation: correlation (top panel) and bland-altman plots (bottom panel) of the measurements extracted from the simulated device and the post-operative device. Each level associated to a different colour (red: ventricular end, blue: nadir, cyan: commissural level, green: central coaptation level.

Table 2 reports the mean differences and coefficients of determination between the measurements extracted from the simulated device and the post-operative device. This is presented for each type of measurement for all levels of the device combined. On every measurement, a great coefficient of determination was achieved (≥0.95). The model slightly underestimated all dimensions, but the mean differences are negligible. Correlation and Bland-Altman plots for each type of measurement are presented in Figure 3, with each level associated to a different colour (red: ventricular end, blue: nadir, cyan: commissural level, green: central coaptation level).

**Table 2:** Mean (±SD) difference between the measurements of the device reconstructed from post-operative DSCT and the simulated devices, and respective coefficient of determination.

Measurement	Mean difference ± SD (postop—simulation)	*R* ^2^
Maximum diameter [mm]	0.02 ± 1.16	0.96
Minimum diameter [mm]	0.80 ± 1.46	0.95
Perimeter [mm]	1.11 ± 2.88	0.98
Area [mm^2^]	14.35 ± 38.48	0.98

### Risk of coronary occlusion analysis

**Figure 4:**
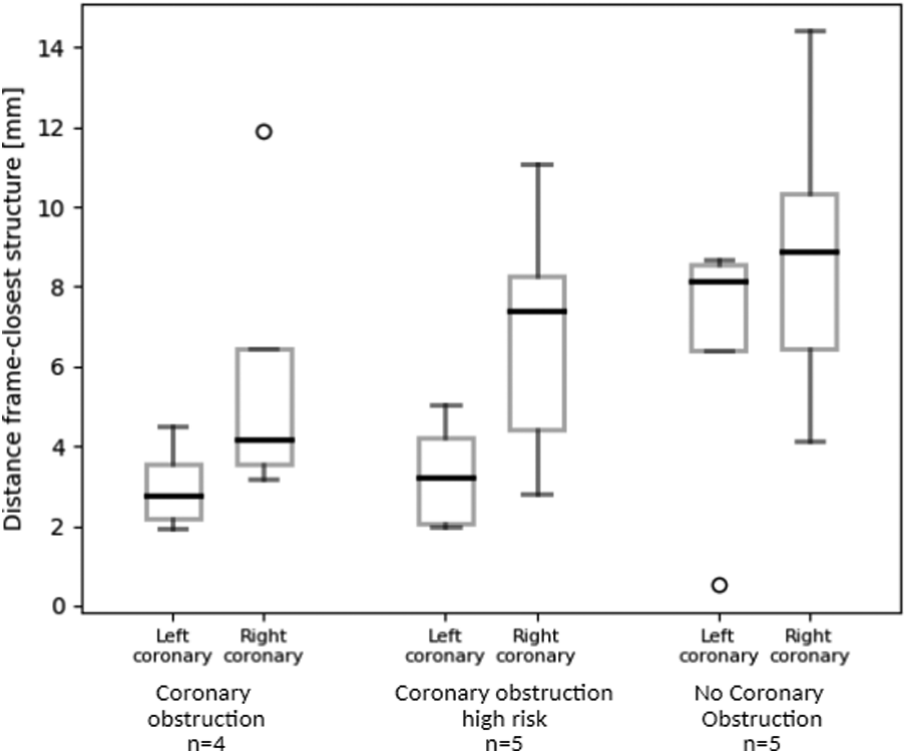
Box plots representation of the simulation-derived distance between the left/right coronary ostium and the closest structure (i.e., native valve leaflets, calcium nodule, deployed Venus-A frame) in patients who experienced CO after TAVR, who were at high risk of CO after TAVR and who had no CO complication.

Simulations predicted shorter distance between the coronary ostium and the closest structure in patients who experienced CO, compared to patients at high risk of CO or who did not experience this complication (right coronary: 5.9 vs. 6.8 vs. 8.8 mm, left coronary: 3.0 vs. 3.3 vs. 6.5 mm respectively). This trend is clearly visible in Figure 4. Also, the distance related to the left coronary was much shorter compared to the right coronary (Table 3). This result nicely relates to the clinical observation (CO or high risk of CO affected the left coronary in all cases).

**Table 3:** Mean (±SD) difference of the predicted distance between the coronary ostia and the closest structure in the stratified subgroups: patients with CO, at high risk of CO, without CO.

Patients with CO (*n* = 4)		Patients at high risk of CO (*n* = 5)		Patients without CO (*n* = 5)	
Left coronary [mm]	Right coronary [mm]	Left coronary [mm]	Right coronary [mm]	Left coronary [mm]	Right coronary [mm]
3.0 ± 1.1	5.9 ± 4.1	3.3 ± 1.3	6.8 ± 3.2	6.5 ± 3.4	8.8 ± 3.9

CO, coronary occlusion.

**Figure 5:**
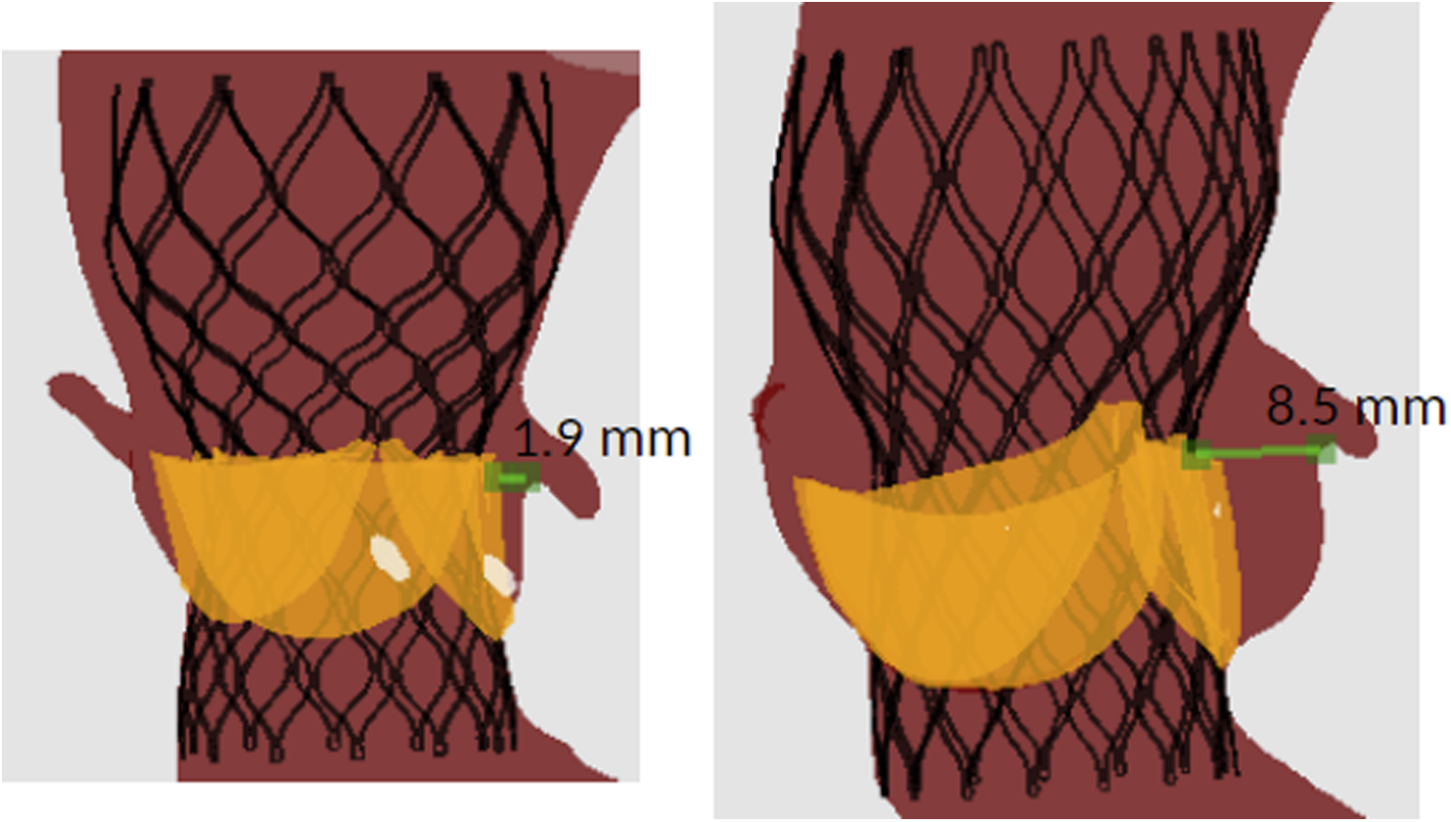
Example of the simulation-derived distance between the left coronary ostium and the closest structure in a patient with (left) and without (right) CO.

Figure 5 shows the distance between the left coronary ostium and the closest structure in a patient with CO (left) and with no CO (right). In both patients the native leaflet tissue was the closest structure to the left coronary ostium. The patient with CO received a Venus-A valve 23 mm (left), while the patient without CO received a Venus-A valve 26 mm (right). The device was implanted at comparable implantation depth.

When considering both coronaries, the native leaflets and the Venus-A frame were the closest structures in the majority of the cases (40% each), while the calcium nodule was close to the coronary ostium in 21% of the cases. Zooming into the subgroup that experienced CO or was at high risk of CO, the blockage was due to calcium nodule or frame apposition in 33% of the cases, while in the majority of the cases (67%) the native leaflets were the closer structures to the coronary ostium, possibly causing full sealing of the sinuses.

## Discussion

This study confirmed the accuracy of the patient-specific simulation predicted frame deformation and found a trend between the minimum distance from the coronary ostia to the closest structure (i.e., native valve, calcium or frame) and the risk of coronary obstruction: the distance decreases with the increase in coronary obstruction risk.

Coronary obstruction is a devastating complication of transcatheter aortic valve replacement (TAVR), with an overall incidence of 0.7% but 30-day mortality of 41% (2). CO generally involves the left coronary artery, which is in line with our cohort (4). The underlying mechanism might be related to the lower distance between the left coronary ostium and annulus plane. Coronary occlusion in TAVR has been a difficult clinical problem to solve, mainly because of the complex structure of the aortic root, the complex morphology of the prosthetic frame, and the complex mechanical interactions between the two.

Meanwhile, several novel techniques were developed in the recent years, such as BASILICA and chimney stenting, which were all proved to be safe and feasible for the prevention of coronary obstruction in patients deemed at high risk. Thus, the most urgent issue that needs to be solved is to efficiently identify patients at risk of coronary obstruction. The virtual transcatheter heart valve (THV) to coronary artery distance (VTC) has been reported to be an indicator for coronary obstruction (10). However, as main limitation, previous studies did not take into account the interaction force between the virtual THV and leaflets, considering the virtual THV in a state without restriction, which is inconsistent with the reality.

Herein we introduce a new analysis using FEops HEARTguide, a patient-specific computer simulation-based analysis of TAVR, to assess the distance between the coronary ostium and the closest structure. Compared to previous studies, the native bulky leaflets are included in the model and their interaction with the implanted Venus-A frame is accounted in the analysis. Our finding clearly shows a trend between such distance and the risk of CO: the distance decreases with the increase in coronary obstruction risk, which is in line with previous findings (4). Next, with FEops HEARTguide is possible to evaluate what structure is causing the CO. Surprisingly, in our cohort the CO (or risk of CO) was mainly caused by the native leaflets which were pushed towards the sinuses, sealing the space to the coronaries (67%). Only in 33% of the cases the blockage was due to calcium nodule or frame apposition. However, previous studies showed that the calcium nodule was the most frequent cause for CO (2, 4). This phenomenon might relate to the lower STJ height of patients with CO (or at risk of CO) in our cohort or the selection bias excluding the patients with high risk of CO potentially caused by heavy calcium nodule.

Assessing the distance between the coronary ostium and the closest structure upfront and evaluating the bulky leaflet displacement due to the implantation of the Venus-A valve upfront, can also support further assessment of the difficulty of future coronary reinterventions. As TAVR progresses to younger patients, the future of coronary reintervention is an issue worth investigating. The distance from the coronary opening to the nearest stent structure shown in our article can be used not only for risk prediction of intraoperative coronary occlusion, but also as a predictor of the accessibility of future coronary intervention. The FEops HEARTguide, as a predictive tool, can help identify patients at higher risk of CO before the TAVI procedure, enabling more informed decision-making about procedural strategies and device selection. In preoperative planning, FEops HEARTguide can assess whether alternative device sizing or positioning could reduce the risk of CO in certain patients, especially those with high risk of CO in anatomical factors.

Some limitations of this study should be listed. First, the sample size is very small, which restricts the generalizability of the results. With limited patients analysed, the statistical power of the study is limited, and the findings may not be representative of the broader patient population undergoing TAVR. This small cohort may have resulted in an underestimation or overestimation of the true predictive capabilities of the simulation models. Additionally, the limited sample size may not fully capture the diversity of anatomical variations and patient-specific factors that can influence outcomes. A cut-off value of the distance between the coronary ostium and the closest structure should be defined to predict the risk for coronary obstruction during preoperative planning. Only the distance between the coronary ostium and the closest structure was investigated in this study. However, there are other parameters, such as the diameter and height of sinotubular junction that could play a role and might be investigated. Finally, fluid dynamic simulation to quantify the flow filling the coronary in diastole could provide additional insight in the grade of possible CO and could be investigated in a follow up study. Conduct studies with larger and more diverse patient populations to enhance the statistical power and generalizability of the findings. Multi-centre study in future could facilitate the recruitment of a broader cohort and provide a more comprehensive evaluation of the simulation tools.

Patient-specific computer simulations offer a powerful tool for enhancing the precision and effectiveness of TAVR procedures. By improving risk stratification, customizing procedural planning, and facilitating informed decision-making, these simulations can significantly impact clinical practice. As technology continues to evolve, the integration of simulations into preoperative planning will likely become a standard component of cardiovascular care, ultimately improving patient outcomes and advancing the field of interventional cardiology.

## Conclusion

The distance between the coronary ostium and the closest structure was lower in patients who experienced CO during TAVR through patient-specific computational simulation. This technology enables coronary obstruction analysis before TAVR in the future.

## Data Availability

The raw data supporting the conclusions of this article will be made available by the authors, without undue reservation.
